# Supplementary material for: A Single Amino Acid Substitution in RFC4 Leads to Endoduplication and Compromised Resistance to DNA Damage in Arabidopsis thaliana
Source: Genes (Basel). 2022 Jun 9;13(6):1037. doi: 10.3390/genes13061037 (PMC9223238; doi:10.3390/genes13061037)
Supplement: Supplementary file 1 [file genes-13-01037-s001.zip › genes-1733933-supplementary.pdf]

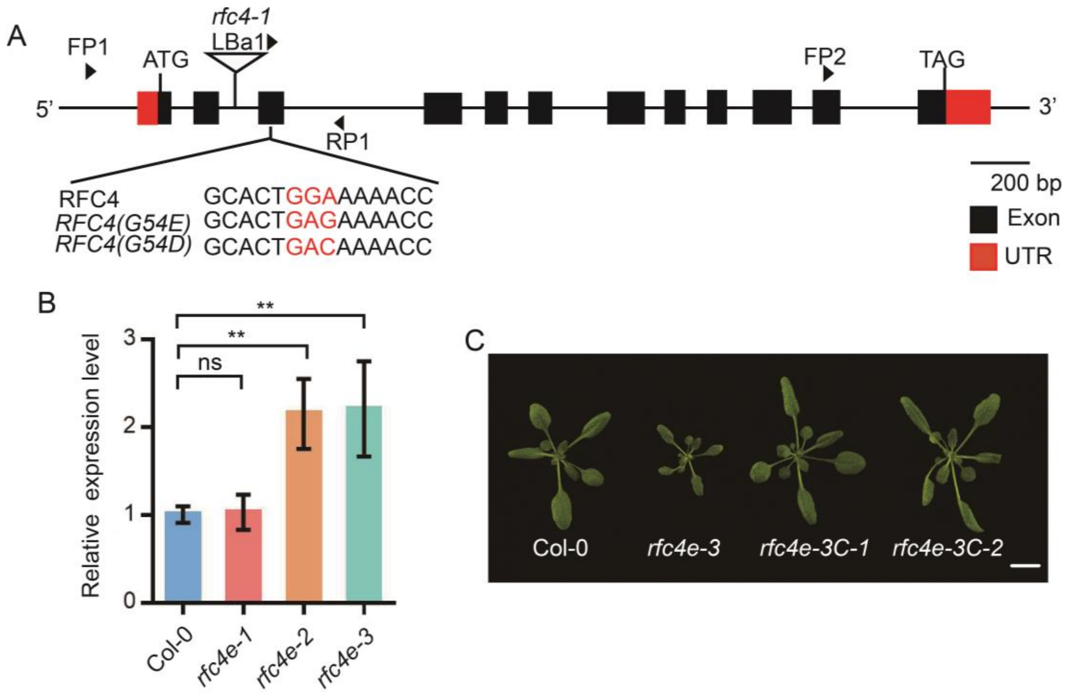

Figure S1. Mutation analysis and complementation assays of *rfc4e-3*. (A) Schematic diagrams of the *AtRFC4* gene and T-DNA insertion position. The black boxes and lines indicate exons and introns, respectively; red boxes represent UTRs and red letters represent mutated bases. (B) The relative expression of *RFC4* or *RFC4* ^{G54E} of Col-0 or *rfc4e-1*, *rfc4e-2* and *rfc4e-3* seedlings at 12 days. Error bars indicate ± SD (***P* < 0.01). (C) Phenotype of wild-type and complementation seedling *rfc4e-3C-1* and *rfc4e-3C-2* at 18 days after germination. Bar = 1 cm.

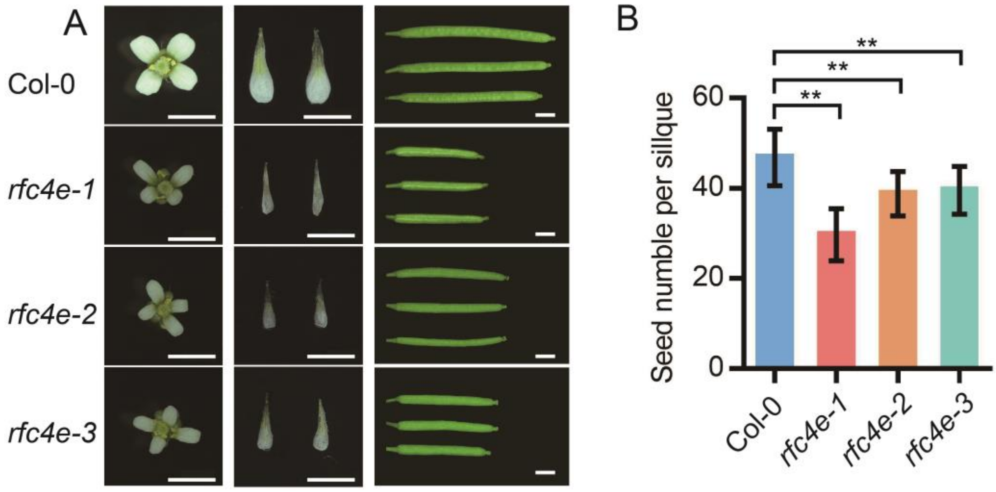

Figure S2. Morphology of representative flowers, petals and siliques for the *rfc4e* mutants. (A) Morphology of representative flowers, petals and siliques of Col-0, *rfc4e-1*, *rfc4e-2* and *rfc4e-3*. Bar = 2 mm. (B) The average number of seeds per silique in Col-0, *rfc4e-1*, *rfc4e-2* and *rfc4e-3*. Error bars indicate ± SD (***P* < 0.01).

Table S1 All DEGs (p < 0.05) analyzed by Gene Ontology (GO) term enrichment based on the biological process category in *rfc4e-3*. The GO enrichment analysis was based on biological process functional categories of ShinyGo v0.61 software, and the top 20 pathways were showed as follows.

Enrichment FDR	nGenes	Pathway Genes	Fold Enrichment	Pathways
9.2E-12	243	1692	1.6	Response to oxygen-containing compound
6.9E-10	187	1265	1.7	Cellular response to stress
3.1E-08	48	195	2.8	DNA replication
1.2E-07	121	770	1.8	Response to radiation
5.6E-07	38	148	2.9	DNA-dependent DNA replication
1.7E-06	220	1716	1.5	Cellular response to chemical stimulus
1.8E-06	73	411	2	Response to water
3.2E-06	33	127	3	Response to karrikin
3.4E-06	54	273	2.3	Response to extracellular stimulus
3.6E-06	44	202	2.5	Response to starvation
3.8E-06	136	962	1.6	Response to inorganic substance
3.9E-06	237	1914	1.4	Response to external stimulus
3.9E-06	46	219	2.4	Cellular response to extracellular stimulus
3.9E-06	34	137	2.8	Double-strand break repair
4.0E-06	75	442	1.9	Response to acid chemical
4.1E-06	66	371	2	Secondary metabolic proc.
6.7E-06	110	746	1.7	Response to light stimulus
8.8E-06	134	966	1.6	Response to lipid
1.5E-05	68	402	1.9	Response to water deprivation
1.6E-05	46	232	2.3	Response to nutrient levels

Table S2 Down-regulated genes (P < 0.05, foldchange ≤ 0.67) analyzed by Gene Ontology (GO) term enrichment based on the biological process category in *rfc4e-3*. The GO enrichment analysis was based on biological process functional categories of ShinyGo v0.61 software, and the top 10 pathways were showed as follows.

Enrichment FDR	nGenes	Pathway Genes	Fold Enrichme nt	Pathway
9.77E-10	22	80	6.826325	Cellular response to phosphate starvation
4.72E-08	26	148	4.360797	Inorganic anion transport
2.78E-08	28	164	4.238073	Cellular response to starvation
1.69E-08	30	179	4.160279	Cellular response to nutrient levels
3.58E-08	31	202	3.80947	Response to starvation
2.74E-08	33	219	3.740452	Cellular response to extracellular stimulus
1.37E-08	71	746	2.362511	Response to light stimulus
2.78E-08	71	770	2.288874	Response to radiation
9.31E-09	128	1716	1.851599	Cellular response to chemical stimulus

Table S3. Up-regulated genes ($P < 0.05$, foldChange ≥ 1.5) analyzed by KEGG term enrichment based on the biological process category in *rfc4e-3*. The KEGG enrichment was analyzed by ShinyGo v0.61 software.

Enrichment FDR	nGenes	Pathway Genes	Fold Enrichment	Pathway
3.88E-08	18	63	6.048823	Homologous recombination
1.05E-05	13	48	5.73378	DNA replication
0.000179	10	37	5.72186	Mismatch repair
0.002875	9	42	4.536617	Base excision repair
0.005244	11	67	3.475816	Nucleotide excision repair

Table S4. Primers used in this study.

Name	Sequence (5′-3′)	Description
FP1	aacaagtaccctacactaaaaggccg	Amplify upstream sequences for *RFC4(G54E)* mutagenesis
At4eMR1	gcagtagtggttttctcagtgcctggtgg	
At4eMF2	ccaccaggcactgagaaaaccactactgc	Amplify downstream sequences for *RFC4(G54E)* mutagenesis
At4R2	gcaatgtccatcaagctatctggtc	
At4F	gaccatgattacgaattcgagctcctacagttttgtatcccagc	Amplified full-length sequence of *AtRFC4*
AT4R	caggtcgactctagaggatccctacactatgatagtctcataag	
RP1	ctgtctagctacctaatggg	*rfc4-1* T-DNA insert identification
LBa1	tggttcacgtagtgggccatcg	
FP1	aacaagtaccctacactaaaaggccg	*AtRFC4* in situ identification
RP1	ctgtctagctacctaatggg	
FLAG-R	aacgatcggggaaattcgag	Site-directed mutagenesis plasmid transfer validation
FP2	agtgacataacagacatgc	
GFP-R	ttcaccctctccactgacag	Complementary plasmid transfer verification
FP2	agtgacataacagacatgc	
AtBRCA1-F	ccatgtattttgcaatgcgtg	At4g21070, for RT-qPCR
AtBRCA1-R	tgtggagcacctcgaatctct	
AtRAD51-F	cgaggaaggatctcttgcag	At5g20850, for RT-qPCR
AtRAD51-R	gcactagtgaaccccagagg	
AtRAD54-F	cagcaggtgacgaggtgac	At3g19210, for RT-qPCR
AtRAD54-R	cgcttatttacaggttggttcc	
AtPARP1-F	acccatcagaggctcaaaca	At2g31320, for RT-qPCR
AtPARP1-R	acgcatcttgatttgttccaca	
AtPARP2-F	tatgccaacactggcgctaa	At4g02390, for RT-qPCR
AtPARP2-R	tcatgtctcccaaagcaacctc	
AtRPA1E-F	tggagaagtgacgactgaagc	At4g19130, for RT-qPCR
AtRPA1E-R	acctccagttgcggaacaat	
AtPCNA1-F	attggaaccgctaaca	At1g07370, for RT-qPCR
AtPCNA1-R	caactccgacgataag	
AtKU80-F	tcgtaaaggctgcgtcttgg	At1g48050, for RT-qPCR

Name	Sequence (5′-3′)	Description
AtKU80-R	ttggcataacttgaatagatggttca	
AtKU70-F	cgagcttcgtgaaaccagagatg	At1g16970, for RT-qPCR
AtKU70-R	ctttcttcatcagggtcatcgcc	
AtPol λ-F	acgacgtgttgaacagaaggc	At1g10520, for RT-qPCR
AtPol λ-R	agctgggaatagtccagtgtcg	
AtLIG4-F	ttggcttcaagtgagaacagagc	At5g57160, for RT-qPCR
AtLIG4-R	tgacccacttcatctcctgagc	
AtNAC053-F	caacagagtttgagccag	At3g10500, for RT-qPCR
AtNAC053-R	ttgccgtatgaccagccca	
AtNAC103-F	gtcactgggaagataagaa	At5g64060, for RT-qPCR
AtNAC103-R	gagaacgcaaagcacataa	
AtGR1-F	gaaggagcagacaaagtgag	At3g24170, for RT-qPCR
AtGR1-R	ggtgagatggaagtgatagg	
AtRAD17-F	ctctggcaagtggagcaatc	At5g66130, for RT-qPCR
AtRAD17-R	tcctcatctgtctcttcgcc	
AtCYCB1;1-F	gatcaatcatcgtcctcgtacacg	At4g37490, for RT-qPCR
AtCYCB1;1-R	cacgtctactacctttggtttccc	
AtSMR4-F	gccgagaagcacgatgtatag	At5g02220, for RT-qPCR
AtSMR4-R	agatctggtggctgaaagtacc	
AtSMR5-F	aaactacgacgacggagatacg	At1g07500, for RT-qPCR
AtSMR5-R	gctaccaccgagaagaacaagt	
AtSMR7-F	gccaaaacatcgattcgggcttc	At3g27630, for RT-qPCR
AtSMR7-R	tcgccgtgggagtgatacaaat	
AtWEE1-F	tattactcctcgtggtt	At1g02970, for RT-qPCR
AtWEE1-R	atgcctttgctatctg	
AtFT-F	gaacaacctttggcaatgagatt	At1g65480, for RT-qPCR
AtFT-R	caccctggtgcatacactgtt	
AtFLC-F	atccgtcgctcttctcgtc	At5g10140, for RT-qPCR
AtFLC-R	cggtcttcttggctctagtca	
AtAP1-F	ccatacaggagcaaaacagc	At1g69120, for RT-qPCR
AtAP1-R	tcttcttgatacagaccacc	
AtACTIN1-F	cgatgaagctcaatccaaacga	At2g37620, for RT-qPCR
AtACTIN1-R	cagagtcgagcacaataccg	
